# A Novel DNA Nanosensor Based on CdSe/ZnS Quantum Dots and Synthesized Fe_3_O_4_ Magnetic Nanoparticles

**DOI:** 10.3390/molecules19044355

**Published:** 2014-04-09

**Authors:** Roozbeh Hushiarian, Nor Azah Yusof, Abdul Halim Abdullah, Shahrul Ainliah Alang Ahmad, Sabo Wada Dutse

**Affiliations:** 1 Institute of Bioscience, Universiti Putra Malaysia, 43400 UPM Serdang, Selangor, Malaysia; E-Mail: hushiarian@gmail.com; 2 Department of Chemistry, Faculty of Science, Universiti Putra Malaysia, 43400 UPM Serdang, Selangor, Malaysia; E-Mails: halim@upm.edu.my (A.H.A.); ainliah@upm.edu.my (S.A.A.A.); swdutse@yahoo.com (S.W.D.); 3 Institute of Advanced Technology, Universiti Putra Malaysia, 43400 UPM Serdang, Selangor, Malaysia; 4 Department of Science Laboratory Technology, HussainiAdamu Federal Polytechnic, Kazaure 705101, Nigeria

**Keywords:** optical DNA nanosensor, DNA hybridization, biosensor, magnetic nanoparticle, quantum dot

## Abstract

Although nanoparticle-enhanced biosensors have been extensively researched, few studies have systematically characterized the roles of nanoparticles in enhancing biosensor functionality. This paper describes a successful new method in which DNA binds directly to iron oxide nanoparticles for use in an optical biosensor. A wide variety of nanoparticles with different properties have found broad application in biosensors because their small physical size presents unique chemical, physical, and electronic properties that are different from those of bulk materials. Of all nanoparticles, magnetic nanoparticles are proving to be a versatile tool, an excellent case in point being in DNA bioassays, where magnetic nanoparticles are often used for optimization of the hybridization and separation of target DNA. A critical step in the successful construction of a DNA biosensor is the efficient attachment of biomolecules to the surface of magnetic nanoparticles. To date, most methods of synthesizing these nanoparticles have led to the formation of hydrophobic particles that require additional surface modifications. As a result, the surface to volume ratio decreases and nonspecific bindings may occur so that the sensitivity and efficiency of the device deteriorates. A new method of large-scale synthesis of iron oxide (Fe_3_O_4_) nanoparticles which results in the magnetite particles being in aqueous phase, was employed in this study. Small modifications were applied to design an optical DNA nanosensor based on sandwich hybridization. Characterization of the synthesized particles was carried out using a variety of techniques and CdSe/ZnS core-shell quantum dots were used as the reporter markers in a spectrofluorophotometer. We showed conclusively that DNA binds to the surface of ironoxide nanoparticles without further surface modifications and that these magnetic nanoparticles can be efficiently utilized as biomolecule carriers in biosensing devices.

## 1. Introduction

A biosensor comprises a biological recognition element, a transducer, and signal processing electronics. The effective bio-recognition site or area interacting with the analyte is a critical parameter that determines the sensitivity of a biosensor and nanoparticles offer promise here because their high surface to volume ratio provides a large number of sites available for molecular immobilization [1].

A magnetic nanoparticle (MNP) is composed of the magnetic cores and a polymeric shell having features favourable for such important applications as environmental remediation, magnetic resonance imaging (MRI), gene and drug delivery systems and cancer therapy [2,3,4,5]. Additionally, the application of MNPs in biosensing has been extensively investigated [6] and a variety of successful techniques have been reported using MNPs in electrochemical DNA and protein sensing [7]. The use of magnetic nanoparticles, especially Fe_3_O_4_ ferrites magnetic nanoparticles has been increasing rapidly because they are superparamagnetic, non-toxic and of a small size. There have been a number of studies using polymers to functionalize and modify the surface of the magnetic nanoparticles [8,9].

Iron oxide magnetic nanoparticles are one of the most important classes of half-metallic materials because of their unique properties. In addition to their high surface to volume ratio, they are highly biocompatible—a property that makes them attractive in biomedicine and research. Furthermore, it is simple to synthesize them and to functionalize their surfaces [10,11,12]. This makes them also of interest for use in the fields of information storage and magnetic sensing [13]. The superparamagnetic behaviour of MNPs is revealed when they are a maximum of 20–30 nm in diameter. As a result of this property, in response to a magnetic field they become easily magnetized and demagnetized. If there is an AC field applied, during the demagnetization they release heat due to loss of energy (specific loss power) [14]. This fact has been exploited for the treatment of cancer through hyperthermia.

Magnetite (Fe_3_O_4_), maghemite (γ-Fe_2_O_3_) and hematite (α-Fe_2_O_3_) are the three naturally occurring forms of iron oxide, the most stable of the three being hematite which is the end product of the transformation of the other two [15]. Compared with hematite, maghemite is metastable, in combination with magnetite forms a continuous solid solution [16] and it has the strongest magnetic properties of all transition metal oxides [15,17]. The detailed crystal structure of these iron oxide forms has been well captured [16,18].

The synthesis of transition metals and their oxides in nano size is eliciting great interest in nanotechnology studies because of the myriad of potential applications [19,20]. The synthesis and characterization of MNPs, in particular, have been investigated extensively because of today’s high demand for compact information storage [16,21]. Some studies focus on developing chemical or physical methods to control the size, shape, morphology and magnetic properties of MNPs [22,23] basing chemical methods on either thermal decomposition of iron compounds in organic solvent or co-precipitation in water using polymers or charged molecules as surfactants [21]. Synthesizing MNPs in organic solvents makes the MNPs remain in an organic phase. It should be noted that they usually include toxic materials among their reactants and consume large amounts of energy [24].

The biological synthesis of MNPs using magneto-tactic bacteria [25,26] and green solvents [27,28] offers an alternative to chemical or physical methods. In biological methods, because they end up in water of neutral pH value at room temperature [29], MNPs synthesized by these methods are being keenly investigated interest for biological applications [30]. The disadvantage is that, due to the hydrophobic surface of MNPs and the high surface area to volume ratio, they usually agglomerate and generate heterogeneous size distribution, leading to rapid clearance from the solution and unexpected results. Thus, it is necessary to disperse them in a suitable solvent or coat them with certain molecules and polymers to form homogeneous solutions called ferrofluids [31].

Ultimately, the surface coating of MNPs is to make more hydrophilic particles using such different strategies as end-grafting, encapsulation, hydrophobic interactions or hyperbranching. Generally different polymers are used for surface modification of magnetic nanoparticles including chitosan, dextran, poly(ethylenimine), poly(ethylene glycol)(PEG) or a copolymer like chitosan-PEG [4]. Surface coating can be carried out during or after synthesis [32]. Surface modification of MNPs can also lead to an increase in biocompatibility and multi-functionality of particles [33]. However, it is known that surface coating results in lowering of the surface to volume ratio and lower magnetic properties [34].

In this study, we successfully designed a simple and inexpensive DNA nanosensor based on MNPs synthesized following a new large-scale method. We made some minor modifications to conventional methods for characterization of the synthesized particles [21,35]. Immobilization of DNA on the surface of the MNPs was achieved by an amide linkage based on the proposed mechanism of surface functionalization of the MNPs during the synthesis procedure.

**Figure 1:**
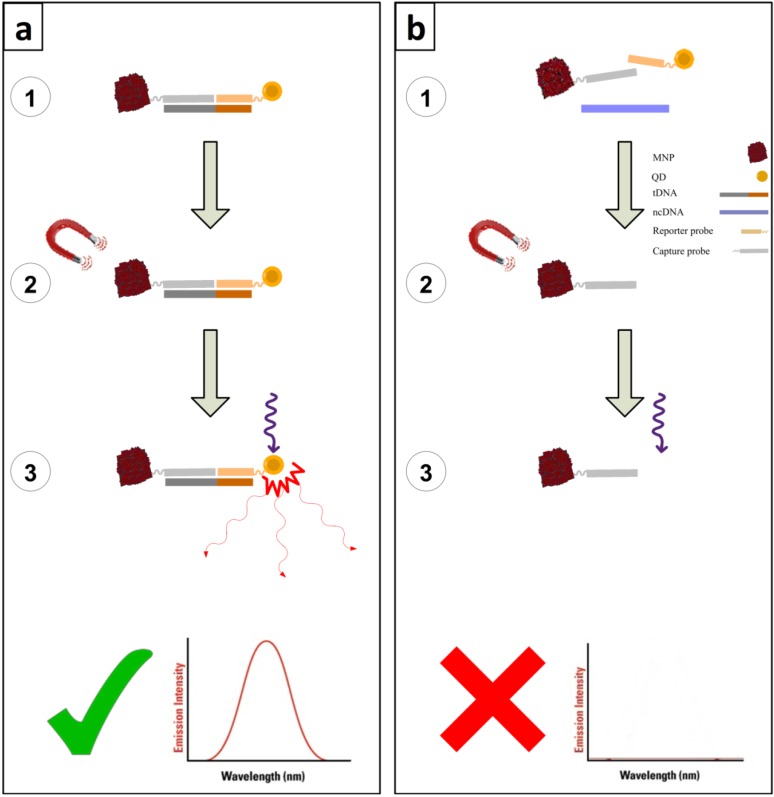
Schematic diagram of the mechanism of the designed optical DNA nanosensor and the related fluorescence spectra. Three different steps of the process are numbered in the diagram. (**a**) In presence of complementary tDNA (**b**) In presence of non-complementary ncDNA.

Figure 1 is a schematic diagram of the designed optical DNA nanosensor based on sandwich hybridization. Figure 1a depicts how the designed sensor works in three steps:

In the first step, reporter probe (_rep_DNA) and capture probe (_cap_DNA) which are bonded to QD and MNP respectively, hybridize to their complementary regions of the target (tDNA). This first step is called hybridization and in this experiment a 10^−5^ M concentration of tDNA was selected.

The second step is called separation and here MNPs are separated based on their magnetic properties and washed by the hybridization buffer. This step is repeated three times so that free molecules and unattached particles are washed out. Thus, by the end of this step, only MNPs and the molecules and particles bonded to them remain in the solution. The sample here contains complementary tDNA.

Finally, using an excitation wavelength of 480 nm, the emission is recorded with the peak at 640 nm indicating that QDs are present in the solution. The blank sample was tested without tDNA and since there was no tDNA to keep _rep_DNA, ultimately the QDs attached to the _cap_DNA-MNP structure were washed out and there was no emission peak while using the 480 nm excitation wavelength. The designed DNA nanosensor was tested with non-complementary (ncDNA) in the absence of tDNA as shown in Figure 1b. In this experiment as there was no complementary region in the ncDNA to the probes attached to the nanoparticles, during the separation step, only MNPs and _cap_DNA attached to them remained in the solution. Thus the results were the same as for the blank in which target DNA was not used.

## 2. Results and Discussion

**Figure 2:**
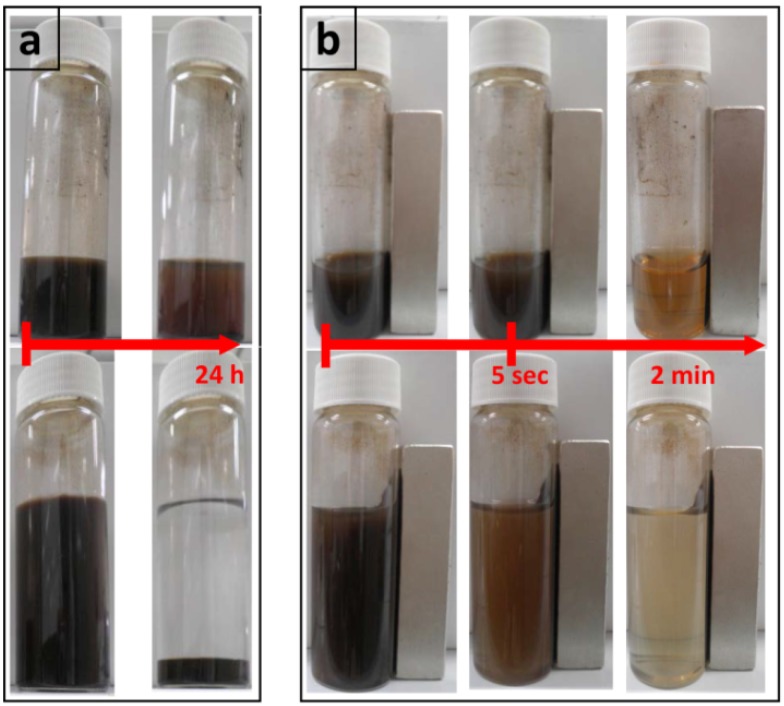
Comparison of stability of MNP suspension in water. Hydrophilic MNPs synthesized in a modified method (**top row**) and in previous chemical method (**bottom row**). (**a**) Samples after 24 h. (**b**) Samples in reaction to 5000 gauss permanent magnet after 5 s and 2 min.

MNPs synthesized in this simple chemical method with some slight modifications showed stronger hydrophilic properties and remained in a water phase for a longer time compared with previously described methods [21,36]. Figure 2 compares the hydrophilicity of MNPs synthesized by the simple chemical method with those of different co-precipitation methods [37]. Figure 2a shows almost no difference in an MNP suspension but after 24 h it is obvious that MNPs synthesized in the modified method are more stable in water (top row) compared with the previous method (lower row) where almost all are precipitated.

In reaction to a 5000 gauss magnet (Figure 2b), hydrophilic MNPs synthesized with the modified method (top row) showed more resistance and a stronger affinity to water. It can be seen that more particles remain in the water after 2 minutes’ absorption by a magnet when they are synthesized in the modified method.

**Figure 3:**
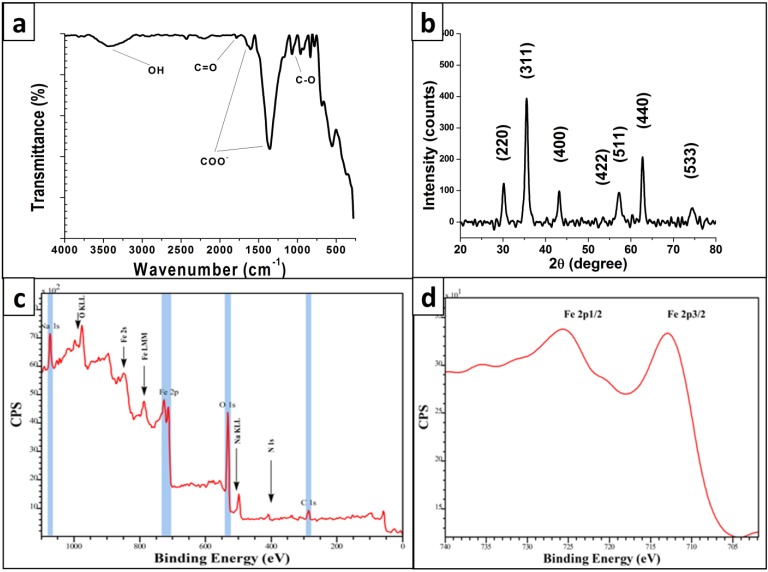
Characterization of structure of synthesized MNPs and their surface functional groups. (**a**) FTIR spectra of synthesized particles; (**b**) XRD pattern; (**c**) XPS of particles and (**d**) The detail of Fe_2p_ peak region in XPS.

The Fourier Transform Infrared (FTIR) spectrum of synthesized MNPs is shown in Figure 3a. The presence of some functional groups on the surface of the particles is obvious. The most significant peaks appeared at 1360 cm^−1^ and 1605 cm^−1^ and are related to the stretching mode of the carboxylic group. This is consistent with previous findings [21]. There are other peaks at 1070 cm^−1^ and 1785 cm^−1^ that are related to C-O and C=O respectively in the carboxylic group. Finally, the broad stretched peak at 3450 cm^−1^ represents OH. The FTIR result clearly suggests that MNPs synthesized in this method are capped by some carboxylic groups which in previous studies were attributed to citrate.

The X-ray diffraction (XRD) pattern of synthesized MNPs (Figure 3b) indicates that magnetite particles have a highly crystalline cubic spinel structure. The diffraction peaks at 30.2, 35.6, 43.2, 53.5, 57.3, 62.8 and 74.4° responded to (220), (311), (400), (422), (511), (440) and (533) planes of cubic Fe_3_O_4_ lattice, respectively. The cubic spinel structure of Fe_3_O_4_ is proven by comparing the XRD pattern with others reported in literature [38,39].

Further investigation of the surface of hydrophilic MNPs was carried out with X-ray photoelectron spectrometry (XPS) and the resultant wide spectra is shown in Figure 3c. Regions of C_1s_, O_1s_ and Fe_2p_ are highlighted in blue indicating that carboxylic groups are present at the surface of the particles. Again, these results are consistent with previous work [21]. After processing the raw data, a closer look at the Fe_2p_ region on the XPS spectrum as depicted in Figure 3d, reveals a peak at 710 eV corresponding to Fe_2p3/2_ and a peak at 723 eV related to Fe_2p1/2_. Values of Fe_3_O_4_ reported in other literature validate these findings [40,41,42,43].

**Figure 4:**
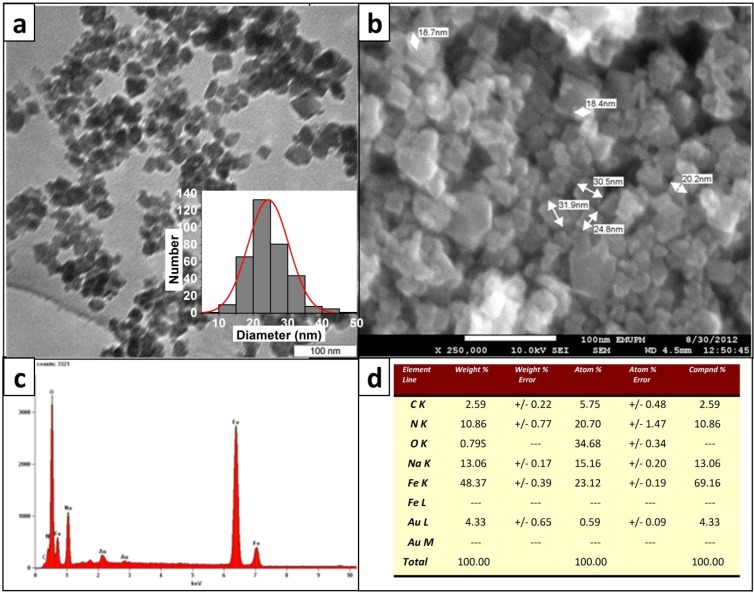
(**a**) TEM image and size distribution of synthesized MNPs. (**b**) FESEM image of the particles. (**c**,**d**) EDX analysis of selected area and its elemental composition.

Transmission electron microscopy (TEM) and Field Emission Scanning Electron Microscopy (FESEM) images shown in Figure 4a,b, respectively, were used to study the size and shape of MNPs. The size distribution of MNPs, illustrated by the histogram, measured three hundred and fifty nanoparticles in different TEM images. More than thirty seven percent of particles were sized between 20 and 25 nm in diameter which is consistent with the size calculation of MNPs using Scherrer’s formula and calculating full width at the half-maximum of major peaks in XRD spectrum of about 20 nm. This suggests that particles are mostly single crystals and confirms previous findings [21].

An energy dispersive X-ray (EDX) analysis of a selected area is shown in Figure 4c. and the elemental composition presented in Figure 4d. Together these results indicate that carbon, oxygen and sodium are major components of MNPs, confirming previous characterization tests and other studies using magnetite [44].

**Figure 5:**
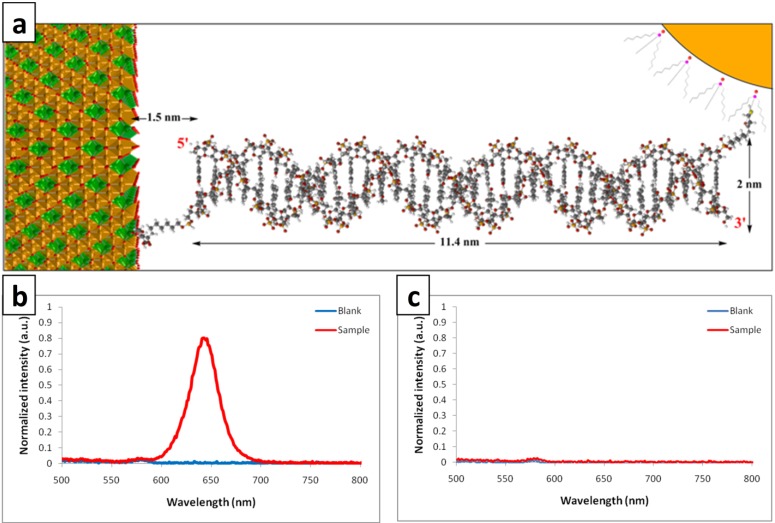
The molecular structure of the designed nanosensor and the related fluorescence spectra. (**a**) molecular model of the designed structure at the presence of tDNA (**b**) Emission spectrum of the constructed system at the presence of complementary tDNA (**c**) Emission spectrum of the system at the presence of non-complementary ncDNA.

Based on characterization tests, immobilization of the capture probe (_cap_DNA) on the surface of the synthesized MNPs was mediated by an amide linkage using EDC and Sulfo-NHS. The same mechanism was employed to immobilize the reporter probe (_rep_DNA) on the surface of QDs. A molecular model of the designed DNA nanosensor at the presence of tDNA is shown in Figure 5a. 5′ and 3′ ends of the tDNA are shown in the figure. Figure 5b is emission spectrum of the designed sensor in spectrofluorophotometer, using an excitation wavelength of 480 nm, the emission is recorded with the peak at 640 nm indicating that QDs are present in the solution after three repeats of separation procedure using permanent magnet. A blank sample was tested without tDNA and related graph is also shown in blue. Since there is no tDNA to keep _rep_DNA, ultimately the QDs attached to the _cap_DNA-MNP structure are washed out and there is no emission peak in the 480 nm excitation wavelength. The same result was seen when ncDNA was used instead of tDNA (Figure 5c). Presence of tDNA in the sample is the key point to keep two other complementary segments together. However, the immobilization of reporter and capture probes on the surface of nanoparticles is crucial to keep QDs attached to the MNPs after separation steps. Successful immobilization of the capture probe on the surface of MNPs confirms the characterization results and the validity of carboxylic groups on the surface of MNPs. Even though the mechanism is not well understood based on a recent study by Parkinson and his colleagues [45] we suggest that “the skyhook effect” in this case induced by citric acid makes the covalent binding of citric acid to the surface of MNPs possible.

## 3. Experimental

### 3.1. Synthesis of Fe_3_O_4_ Magnetic Nanoparticles

The modified chemical process followed to synthesize the MNPs was as follows: firstly, a mixture of sodium hydroxide (NaOH, 4 mmol), sodium nitrate (NaNO3, 0.2 mol) and sodium citrate (C_6_H_5_Na_3_O_7_·2H_2_O, 1 mmol) in distilled water (19 mL) was prepared and heated to form a pellucid solution. a preheated solution of 2 M FeSO_4_^.^4H_2_O (1 mL, 2 mmol) was quickly added into the mixture and kept at 100 °C for 45 min. The solution was shaken several times, whilst it was held at 100 °C. Next, the solution was cooled on ice and then the synthesized MNPs were separated from the solvent using a 5000 gauss permanent magnet. Finally, the synthesized MNPs were washed several times. The end product, hydrophilic MNP powder (Fe_3_O_4_), will keep indefinitely but takes only a few minutes to be re-dispersed in an ultrasonic water bath when required for re-use.

### 3.2. Characterization of MNPs

A range of techniques was used to characterize the synthesized MNPs. X-ray diffraction (XRD) was carried out with Cu Kα radiation (λ = 1.54056 Å) generated at 40 kV and 30 mA using Shimadzu XRD-6000 to produce diffraction patterns from crystalline powder and determine the structure. The spectrum was continuously scanned in the range of 2θ = 10–90° and at a rate of 2°/min. X-ray photoelectron spectrometry (XPS) employing a Kratos/Shimadzu Axis ultra DLD was used to measure the surface of MNPs. The presence of functional groups, particularly carboxylic group, on the surface of MNPs was investigated utilizing Fourier Transform Infrared Spectroscopy (FTIR): Perkin Elmer Spectrum 100. Transmission electron microscopy (TEM) and Field Emission Scanning Electron Microscopy (FESEM), with Hitachi H7100 and JEOL JSM-6400 models, respectively, were employed for imaging the synthesized MNPs. For TEM, a solution of MNPs diluted in water was kept in an ultrasonic water bath for 15 min and then a droplet of solution put on a copper grid holder. Imaging was done after drying out the water. For FESEM, dried MNP powder was attached to a holder using carbon paint. An analysis of the elements and chemical characterization of the sample was obtained by Energy-Dispersive X-ray spectroscopy (EDX) at a voltage of 20 kV using a Hitachi S-3400N instrument. The MNP powder was dispersed on a copper holder. Using a double-sided carbon tape the powder was attached to the holder. The powder was then coated with gold using a BIO-RAS Sputter Coater.

### 3.3. Nucleic Acid Sequences

A 35 base ssDNA was named as the target (tDNA) (5′-TTG GCT CTC GCA TCG ATG AAG AAG AAC GCA GCA GG-3′) was carefully chosen from a ribosomal RNA (rRNA) gene of *Ganoderma boninense*, a major pathogen of oil palm trees [46]. A 20 base oligonucleotide complementary to the 3′end of the target DNA was designed and named as the capture probe (_cap_DNA) (5′-/5AmMC_6_/CCT GCT GCG TTC TTCTTC AT-3′). A 15 base oligonucleotide complementary to the 5′end of the target DNA was designed and named as the reporter probe (_rep_DNA) (5′-CGA TGC GAG AGC CAA/3AmMC_6_/-3′). The Amine group at the 5′end of the capture probe and the 3′end of the reporter probe was applied for later binding through amide linkage to the surface of the nanoparticles. A 35 base ssDNA with the sequence of (5′-GGA AGG CCA GCT ACA ACC CAG CTA GTC AAG GTA AC-3′) was used in the experiment as non-complementary DNA (ncDNA). The designed DNA sequences were synthesized by 1st BASED Laboratories Sdn Bhd, Selangor, Malaysia.

### 3.4. Surface Modification of Nanoparticles

Surface modification of quantum dot (QD) nanoparticles was conducted using a well-known protocol [47] with some small modifications. Trioctylphosphine oxide (TOPO, 500 µL)-protected Lumidot^TM^ CdSe/ZnS nanoparticles in toluene, purchased from Sigma-Aldrich (Saint Louis, MO, USA) was incubated with amphipatic molecule mercaptopropionic acid (MPA, 100 µL) overnight. The whole reaction was covered with aluminum foil. By adding KOH solution (1 M, 1 mL), the nanoparticles were transferred to the water phase. Uncoated QD nanoparticles were washed out using toluene (1 mL). The remaining MPA molecules were washed out by three repeats of the QD nanoparticle precipitation procedure and re-dissolution in phosphate buffer solution (10 mM) at pH 7.4.

### 3.5. Immobilization of DNA Sequences

The immobilization of the 15-mer reporter DNA probe onto the surface of QDs was carried out by a previously reported method [48]. Briefly, a mixture of QDs (0.05 µM, 40 µL), 1-ethyl-3-[3-dimethylaminopropyl] carbodiimide hydrochloride (EDC, 2.6 mM, 20 µL) and sulfo-*N*-hydroxysuccinimide (Sulfo-NHS, 4.3 mM, 20 µL) in water with pH 6.0 was prepared and incubated at 37 °C for one hour. At this stage the carboxylic group of MPA on the surface of QDs became activated. Subsequently, the 3′-amine capped reporter DNA probe (10 µM, 10 µL) (_rep_DNA/QDs = 50:1) was added and the whole solution was incubated for another one hour at 37 °C. During this stage DNA covalently binds to MPA via an amide bond. These QDs with immobilized DNA on them are estimated to be stable for over two months at 4 °C when kept in aluminum foil.

Since it is proposed that MNPs synthesized in this method contain carboxylic groups on their surface, the procedure was repeated to bind the 20-mer 5′aminated capture DNA probe onto the surface of MNPs via amide bond.

### 3.6. DNA Hybridization

The hybridization experiment followed a described protocol [49] with some changes appropriate to this experiment and some modifications due to the sequences. It was performed simply in a buffer solution containing 100 mM Tris-HCl, 10 mM (NH_4_)_2_SO_4_ and 3 mM MgCl_2_ with pH 8.0. All the participants of hybridization, including QD-_rep_DNA, MB-_cap_DNA and tDNA, were incubated in a reaction buffer for 30 min at 52 °C. After cooling to room temperature, MNPs were absorbed using a 5,000 gauss permanent magnet and supernatant was discharged. Next, the MNPs were re-dispersed in water and this washing procedure was repeated three times. Finally, the fluorescence spectra were captured with a Shimadzu RF-5301 PC spectrofluorophotometer.

## 4. Conclusions

While there have been studies such as those described by Dong [50] and Sharon [51] which have reported the use of either ferromagnetic nanoparticles or fluorescent quantum dots for DNA sensing, this study is unique in its integration of both techniques. The essence of the novel aspect of our work was our experimentation with binding DNA to the surface of the MNPs without surface modification. In summary, MNPs synthesize in a simple chemical method dispersed in water very well, due to the existence of carboxylic groups on their surfaces. What we did here was to experiment using MNPs as biomolecular carriers and immobilizing DNA on their surfaces via an amide linkage. We were then able to design and test an optical DNA nanosensor based on sandwich hybridization and show that it was capable of distinguishing tDNA from ncDNA. Our goal was successfully achieved.

In the meantime there continue to be many other methods of exploiting the features of MNPs. Wei Wu and colleagues [52] summarize three major strengths of the many current strategies for obtaining functionalized magnetic iron oxide NPs:

1. Improve the biocompatibility and chemical stability, and tailor the dispersability and water solubility.
2. Endow the iron oxide new physico-chemical properties, such as magnetic-optical properties, magnetic-electrical properties, magnetic-thermal properties, *etc*.
3. Provide the iron oxide new functional end groups for the subsequent functionalized procedures or the subsequent applications, such as conjugation with the DNA, antibody, protein, *etc*.

In research with ‘live’ samples, because DNA is double stranded and enclosed inside the pathogen cells, there are always challenges with proper sampling, extraction and denaturation of the DNA prior to the detection. We also faced some issues working with MNPs because this required separation using a magnet and also repeated washing steps which impacted detrimentally on the efficiency and final yield of the process. Finally, we had some difficulties working with the DNA as the probes we designed for detection of the pathogen were complementary to only a very limited and short sequence of the total DNA. This meant that there was a high probability of nonspecific bindings and mismatches under varying conditions.

Indeed, more generally, researchers still need a better understanding of how to synthesize high-quality functionalized magnetic iron oxide NPs in a controlled manner, how to improve their stability and availability in extreme environmental conditions, how to develop efficient and orderly magnetic nano-assembly structures, and how to realize large-scale or industrial synthesis.

There is no doubt that the surface functionalization and modification of magnetic iron oxide NPs will continue to be studied globally by many scientists for such applications as magnetic recording, bioseparation, biodetection, MRI, gene and drug delivery systems as well as cancer therapy, contributing to new knowledge in nanobiotechnology and ultimately to the improvement of our quality of life.
